# CRABPs Alter *all-trans*-Retinoic Acid Metabolism by CYP26A1 via Protein-Protein Interactions

**DOI:** 10.3390/nu14091784

**Published:** 2022-04-24

**Authors:** King Clyde B. Yabut, Nina Isoherranen

**Affiliations:** Department of Pharmaceutics, School of Pharmacy, University of Washington, Seattle, WA 98195, USA; yabutk@uw.edu

**Keywords:** cellular retinoic acid binding protein, fatty acid-binding protein, cytochrome P450 26, *all-trans*-retinoic acid, vitamin A metabolism, protein–protein interactions, free drug hypothesis

## Abstract

Cellular retinoic acid binding proteins (CRABP1 and CRABP2) bind *all-trans*-retinoic acid (*at*RA), the active metabolite of vitamin A, with high affinity. CRABP1 and CRABP2 have been shown to interact with the *at*RA-clearing cytochrome P450 enzymes CYP26B1 and CYP26C1 and with nuclear retinoic acid receptors (RARs). We hypothesized that CRABP1 and CRABP2 also alter *at*RA metabolism and clearance by CYP26A1, the third key *at*RA-metabolizing enzyme in the CYP26 family. Based on stopped-flow experiments, *at*RA bound CRABP1 and CRABP2 with K_d_ values of 4.7 nM and 7.6 nM, respectively. The unbound *at*RA K_m_ values for 4-OH-*at*RA formation by CYP26A1 were 4.7 ± 0.8 nM with *at*RA, 6.8 ± 1.7 nM with holo-CRABP1 and 6.1 ± 2.7 nM with holo-CRABP2 as a substrate. In comparison, the apparent k_cat_ value was about 30% lower (0.71 ± 0.07 min^−1^ for holo-CRABP1 and 0.75 ± 0.09 min^−1^ for holo-CRABP2) in the presence of CRABPs than with free *at*RA (1.07 ± 0.08 min^−1^). In addition, increasing concentrations in apo-CRABPs decreased the 4-OH-*at*RA formation rates by CYP26A1. Kinetic analyses suggest that apo-CRABP1 and apo-CRABP2 inhibit CYP26A1 (K_i_ = 0.39 nM and 0.53 nM, respectively) and holo-CRABPs channel *at*RA for metabolism by CYP26A1. These data suggest that CRABPs play a critical role in modulating *at*RA metabolism and cellular *at*RA concentrations.

## 1. Introduction

*all-trans*-retinoic acid (*at*RA) is the active metabolite of vitamin A (retinol) and is essential for a variety of physiological processes critical for life. The biological activity of *at*RA is mediated through *at*RA binding to its canonical nuclear receptors, retinoic acid receptors (RARs). RAR signaling regulates cell growth and differentiation and cell cycle progression during embryonic development and adult life [1,2,3,4]. As RARs are ligand activated nuclear receptors, RAR activation and signaling are regulated by changing the cellular concentrations of *at*RA [5,6,7,8,9,10,11,12]. For example, postnatal, conditional knock out of the major *at*RA-clearing cytochrome P450s (CYPs) Cyp26a1 and Cyp26b1 in mice results in increased *at*RA concentrations and aberrant physiology in the spleen and skin [5]. Similarly, hematopoietic stem cell (HSC) differentiation and renewal is controlled by changes in *at*RA signaling and metabolism. Inhibition of *at*RA synthesizing enzymes promotes HSC self-renewal, while treatment with *at*RA reverses these effects, driving HSCs toward differentiation [13]. HSC differentiation was also increased with the inhibition of *at*RA*-*metabolizing enzymes in the bone marrow stroma [2], demonstrating that synthesis and metabolism of *at*RA, likely via altering local *at*RA concentrations, control retinoid signaling.

The cellular concentrations of *at*RA are regulated by a network of metabolic enzymes including the CYP26 family of enzymes that oxidize *at*RA to 4-OH-*at*RA and are the main enzymes clearing *at*RA in all chordates [14,15,16]. In addition to the CYP26 enzymes (CYP26A1, CYP26B1 and CYP26C1 [17,18,19]), other adult and fetal liver CYP enzymes such as CYP3A4, CYP3A7 and CYP2C8 also form 4-OH-*at*RA and may have a role in *at*RA clearance. Yet, their quantitative role in modulating *at*RA concentrations is not well defined [20]. The two major CYP26 enzymes CYP26A1 and CYP26B1 both metabolize *at*RA to 4-OH-*at*RA with high efficiency and the biochemical function of the two enzymes is similar [18]. However, both CYP26A1 and CYP26B1 are essential for embryonic development and the two enzymes cannot compensate for each other in vivo [1,14]. The expression patterns of CYP26A1 and CYP26B1 are distinct both during embryonic development and in adult animals [14]. CYP26B1 appears to be the high-affinity, low-capacity CYP26 enzyme while CYP26A1 has a higher capacity and is a lower-affinity enzyme for *at*RA clearance [18,21]. CYP26A1 is also the predominant CYP26 in the adult liver and likely the main enzyme clearing exogenous *at*RA [21]. In contrast, CYP26B1 is primarily found in extra-hepatic tissues [5,14]. Overall, both CYP26A1 and CYP26B1 are needed for *at*RA metabolism and regulate tissue *at*RA concentrations, but their tissue-specific functions remain to be fully elucidated.

Vitamin A homeostasis is affected by a number of cellular binding proteins that impact retinol esterification, retinyl ester hydrolysis, retinol, retinaldehyde and *at*RA oxidation, and trafficking of retinoids to different cellular compartments and tissues [22]. Activation of nuclear receptors by *at*RA also appears affected by specific interactions with cellular retinoic acid binding proteins (CRABP1 and CRABP2). CRABPs are intracellular retinoid binding proteins that bind *at*RA with high affinity [23,24,25,26,27,28] and *at*RA is likely sequestered by CRABPs in cells in which CRABPs are expressed. To coordinate *at*RA signaling, CRABPs have been proposed to have distinct roles in delivering *at*RA to nuclear receptors, with CRABP2 directly channeling *at*RA to RARα [24,29,30]. Studies in COS-7 cells showed that apo-CRABP2, anchored to the endoplasmic reticulum (ER), dissociates from the ER upon binding to *at*RA and translocates into the nucleus to activate RAR signaling via a SUMOylation dependent mechanism [30] prior to returning to the ER. On the other hand, holo-CRABP1 did not translocate to the nucleus to activate RARs [24]. These data suggest that CRABPs play an important role in modulating *at*RA signaling and delivery to target proteins and enzymes. In addition to CRABPs, epidermal fatty acid-binding protein (FABP5) also appears to bind *at*RA and subsequently translocate to the nucleus to drive *at*RA signaling towards cell proliferation genes through PPARβ/δ activation [29], but the broader role of FABP5 in retinoid signaling has not been defined.

In addition to channeling *at*RA to nuclear receptors and regulating RAR activation, CRABPs may also channel *at*RA to metabolic enzymes or alter *at*RA clearance [15,16,19,22,31,32]. Early studies in rat testis microsomes found that *at*RA was metabolized at similar rates in the presence and absence of CRABP1, while increasing CRABP1:*at*RA ratio to 3:1 resulted in decreased k_cat_ for *at*RA oxidation. These data could not be explained by the free drug hypothesis and suggest that holo-CRABP may channel *at*RA for metabolism [15,16]. Indeed, subsequent metabolic studies of *at*RA with CYP26B1 and CYP26C1 in the presence of CRABPs showed that the 4-OH-*at*RA formation kinetics in the presence of CRABPs can only be explained by direct protein–protein interactions between CYP26B1/C1 and CRABPs, and likely substrate channeling from holo-CRABPs to CYP26s. The apparent K_m_ values of *at*RA bound to CRABPs with CYP26B1 and CYP26C1 were lower or similar to the K_m_ values observed in incubations with *at*RA alone, and the k_cat_ values were decreased in the presence of CRABPs [19,31]. This is contrary to expectations based on the free drug hypothesis, where apparent K_m_ values should increase when *at*RA is bound to CRABPs with no effect on the k_cat_ [19,31]. Kinetic modeling of the metabolism of *at*RA by CYP26B1 in the presence of CRABPs suggested a direct protein–protein interaction between CYP26B1 and CRABPs. Similar observations suggesting that holo-CRABP2 is also accepted as a substrate for CYP27C1, a retinoid desaturase in the skin, were recently reported [32]. Whether similar protein–protein interactions occur between CYP26A1 and CRABPs has not been established.

We hypothesized that CRABP1, CRAPB2, and FABP5 bind *at*RA with high affinity and alter the metabolism of *at*RA by CYP26A1 via direct protein–protein interactions. The goal of this study was to determine if CRABP1, CRABP2 or FABP5 have an impact on *at*RA metabolism by CYP26A1 and to elucidate the role that intracellular lipid binding proteins have in regulating *at*RA metabolism through CYP26 enzymes. Our data show that CRABP1 and CRABP2 play a role in modulating cellular *at*RA homeostasis and that the apo- to holo-CRABP ratio has a unique potential role in regulating retinoid concentrations, metabolism and signaling.

## 2. Materials and Methods

### 2.1. Chemicals and Reagents

Kanamycin, sodium chloride, potassium chloride, Tris, Hepes, potassium phosphate, glycerol, benzonase, lysozyme, thrombin, protease inhibitor tablets, Coomasssie Brilliant Blue and *at*RA were purchased from Millipore-Sigma (St. Louis, MO, USA). The 4-oxo-*at*RA-d_3_ was purchased from Santa Cruz Biotechnology (Dallas, TX, USA) and 4-OH-*at*RA was purchased from Toronto Research Chemicals (North York, ON, Canada). Sodium hydroxide, PMSF, imidazole, Pierce BCA protein assay, DTT, EDTA, IPTG, tryptone, yeast extract, high-performance liquid chromatography (HPLC) and mass spectrometry grade acetonitrile, water, and ethyl acetate were purchased from Thermo Fisher Scientific (Waltham, MA, USA). Lipidex-5000 was purchased from Perkin Elmer Inc (Waltham, MA, USA). Mini-PROTEAN TGX protein gels were purchased from Bio-Rad (Hercules, CA, USA). NBD-stearate was purchased from Avanti Polar Lipids (Birmingham, AL, USA). Pooled human liver microsomes were purchased from Gibco (Thermo Fisher Scientific, Waltham, MA, USA). Recombinant CYP3A4 and CYP2C8 Supersomes co-expressed with cytochrome P450 reductase and cytochrome b5 were purchased from Corning (Glendale, AZ, USA). Recombinant CYP26A1 was expressed in Sf9 insect cells, as previously described [17,20]. Rat P450 reductase was expressed in *E. coli* and purified, as previously described [33].

### 2.2. Expression and Purification of CRABP1 and CRABP2

CRABP1 and CRABP2 expression constructs were a gift from Noa Noy (Case Western Reserve University). These CRABP1 and CRABP2 clones, in a pET28a vector, contained a thrombin cleavage site and an N-terminal hexa-histidine (6×his) tag. CRABP1 contained an additional N-terminal T7 tag (Figure S1) The plasmid map and sequences for CRABP1 and CRABP2 are shown in Supplementary Materials. Both proteins were expressed in Rosetta 2 *E. coli* (Novagen, Madison, WI). A 25 mL starter culture with a freshly transformed colony in LB-kanamycin (50 μg/mL) was grown for 6 h at 37 °C in a shaking incubator at 250 RPM. A total of 15 mL of the starter culture was used to inoculate 1 L of LB-kanamycin which was grown to an OD600 ≈ 0.6. The culture was cooled to room temperature over 20 min prior to adding 0.1 mM IPTG to induce CRABP1 and CRABP2 expression. The proteins were expressed at 18 °C for 18 h in a shaking incubator at 220 RPM. Cells were harvested by centrifugation at 5000 *g* for 20 min, washed with PBS containing 1 mM PMSF, pelleted, decanted and stored at −80 °C.

Frozen pellets were thawed on ice in 25 mL of lysis buffer per one L of culture (20 mM Tris pH 7.4, 500 mM NaCl and 30 mM imidazole) with protease inhibitor cocktail (Roche, cOmplete Mini EDTA-free), 1 mM PMSF and 25 U of benzonase. A total of 1 mg/mL lysozyme was added to the resuspended cells and the suspension was rocked on ice for 20 min. To ensure complete lysis, cells were sonicated at 75% power for 30 s with a 1 min rest on ice (5 rounds). The lysate was cleared via centrifugation at 20,000 *g* for 30 min and supernatant filtered through a 0.45 μm Amicon syringe filter (Milipore-Sigma, St. Louis, MO, USA). The filtered lysate was loaded onto a 90 mL Dynaloop (Biorad, Hercules, CA, USA) coupled to a DuoFlow fast protein liquid chromatography (FPLC) (Bio-Rad, Hercules, CA, USA) and a 1 mL HisTrap affinity column (GE Healthcare, Chicago, IL, USA), equilibrated in lysis buffer at flow rate of 15 mL/min. For protein purification, the column was washed with 10 column volumes of wash buffer (20 mM Tris pH 7.4, 500 mM NaCl, 30 mM imidazole) and CRABPs were eluted with 300 mM imidazole in elution buffer (20 mM Tris pH 7.4, 500 mM NaCl) in 1 mL fractions over 10 column volumes. Protein elution was detected at 280 nm absorbance and the CRABP-containing fractions combined. The eluted protein was concentrated, and buffer exchanged into thrombin cleavage buffer (20 mM Tris, pH 7.4, 500 mM NaCl) using a 10 kDa molecular weight cutoff (MWCO) Amicon concentrator (Milipore-Sigma, St. Louis, MO, USA). After buffer exchange, the protein concentration was measured by Nanodrop (Thermo Fisher, Waltham, MA, USA) by absorbance at 280 nm. The N-terminal 6×his tag was cleaved by adding 0.03 U of thrombin protease per 10 μg of CRABP into the solution and incubating overnight (>12 h) at 4 °C. Cleavage of the N-terminal tag was confirmed via SDS-PAGE with Coomassie staining and an anti-his (mouse anti-His antibody from Qiagen, Valencia, CA, USA) Western blot. The cleaved protein was then injected into a Superdex 75 size exclusion column (GE Healthcare, Chicago, IL, USA) equilibrated with HEK buffer (10 mM Hepes, pH 8, 100 mM KCl, 0.1 mM EDTA) using DuoFlow FPLC at a flow rate of 0.5 mL/min. After injection, the flow rate of the HEK buffer was increased to 1 mL/min and the CRABP-containing fractions, as detected at 280 nm, were collected, pooled and concentrated using a 10 kDa MWCO Amicon concentrator. The final concentration of CRABPs was quantified by a BCA protein assay. DTT (0.5 mM final concentration) and glycerol (50% final concentration) were added to the protein solution in HEK buffer and the CRABPs were stored at −20 °C. The binding of *at*RA to the CRABPs was verified via fluorescence titrations, as previously described [23]. Briefly, titrations were prepared in a 96-well black-walled plate with 2 µM CRABP and 0–2.8 µM *at*RA in 100 mM potassium phosphate, pH 7 under protection from light. The quenching of CRABP tryptophan fluorescence (excitation at 290 nm and emission at 340 nm) by *at*RA binding was measured at 37 °C with a Gemini Em fluorescence plate reader (Molecular Devices, San Jose, CA, USA) with the emission auto-cutoff set to on. Endpoint fluorescence scans were carried out with the photomultiplier tube (PMT) sensitivity set to auto with 6 reads and auto-calibration and auto-mixing for 2 s.

### 2.3. Expression and Purification of FABP5

Human FABP5 from OriGene (Rockville, MD, Cat. No. SC119223) was amplified with an N-terminal NdeI restriction site and a C-terminal HindIII restriction site and subcloned into pET28a with a 6×his tag and thrombin cleavage site (Figure S1). Constructs were restriction-digested and sequence-verified and FABP5 was expressed in Rosetta 2 cells under the same conditions as described above for CRABPs.

FABP5 was purified using the method described above for CRABPs with the following changes: 50 mM Hepes pH 7.2, 250 mM NaCl, 30 mM imidazole was used as lysis and wash buffers, and FABP5 was eluted with a stepwise imidazole gradient (30–500 mM) over 10 column volumes. The eluted FABP5-containing fractions were pooled, concentrated using an Amicon concentrator with a 10 kDa MWCO and injected into a Superdex 75 column equilibrated with gel filtration buffer (30 mM Tris, pH 7.6, 100 mM NaCl), coupled to a DuoFlow FPLC at a flow rate of 0.5 mL/min. Fractions containing FABP5 were collected, pooled and passed five times through a 5 mL Lipidex-5000 gravity flow column equilibrated with gel filtration buffer to remove any contaminating *E. coli* lipids from the FABP5. Delipidated FABP5 was concentrated using an Amicon concentrator (10 kDa MWCO), flash frozen and stored in −80 °C in 30 mM Tris, pH 7.6, 100 mM NaCl. FABP5 protein concentration was quantified via a BCA protein assay and NBD-stearate binding to FABP5 was confirmed via fluorescence, as described previously [34].

### 2.4. Preparation of CYP26A1 Microsomes from Sf9 Insect Cells

To prepare CYP26A1 microsomes, insect cell pellets containing expressed recombinant CYP26A1 [17] were resuspended in homogenization buffer (10 mM potassium phosphate, 0.25 M sucrose, 0.2 mM PMSF, pH 7.4) and sonicated for 10 s with a 30 min rest on ice for 3 rounds. The cells were further lysed with one pass through a Thomas tube and a Teflon pestle attached to a hand power drill. The homogenized cells were centrifuged at 20,000 *g* for 20 min to remove cellular debris, nuclei and other large organelles. The supernatant was collected and centrifuged at 100,000 *g* for 60 min to pellet the microsomal fraction (ER membrane fragments). After decanting, the pellet was rinsed with storage buffer (50 mM potassium phosphate, 0.1 mM EDTA, 20% glycerol, pH 7.4) and resuspended in 2 mL of storage buffer, flash frozen in liquid nitrogen and stored at −80 °C. The concentration of CYP26A1 was determined via a CO-difference spectrum [35].

### 2.5. Determination of atRA-Binding Kinetics with CRABP1 and CRABP2 by Stopped-Flow

Binding kinetics of *at*RA with CRABP1 and CRABP2 were measured using an SX20 stopped-flow instrument (Applied Photophysics, Leatherhead, Surrey, UK) with a 1 ms dead time. A 2 mm optical pathlength was used and the detector voltage was set to 300 V. The excitation wavelength was set to 350 nm and the increase in *at*RA fluorescence due to binding to CRABPs was monitored with a 455 nm emission cut-off filter. All binding experiments were carried out at room temperature and protected from light. To measure *at*RA-binding kinetics with CRABP1 and CRABP2, separate solutions of 1 µM CRABP1 or CRABP2 and 4 μM *at*RA were prepared in an assay buffer in amber glass vials, and CRABP and *at*RA solutions were loaded into separate stopped-flow drive syringes. After injection, the final mixed concentrations were 0.5 µM CRABP and 2 μM *at*RA. Data were acquired for 2 sec with 1000 measurements per injection. Replicate experiments were conducted on three different days and each replicate experiment consisted of 5 subsequent injections.

To determine the concentrations of observed holo-CRABP in stopped-flow experiments, the fluorescence per mol of holo-CRABP was measured. The steady state fluorescence of holo-CRABP1 and holo-CRABP2 was measured at a final mixed concentration of 0.5 μM of CRABP and 2 and 5 μM of *at*RA. Two different concentrations of *at*RA in excess were used to ensure saturation of CRABP binding. The fluorescence yield of holo-CRABP was calculated from the average fluorescence between 0.4 and 0.8 s, and 0.6 and 1.0 s for holo-CRABP1 and holo-CRABP2, respectively. This average fluorescence from all experiments was divided by the total concentration of CRABP (0.5 μM) to obtain the value of CRABP fluorescence per mol of CRABP. The fluorescence per mol of holo-CRABP determined above was used to calculate the concentrations of observed holo-CRABP1 or holo-CRABP2 in kinetic stopped-flow experiments and a 2-state kinetic binding model was fitted to the observed holo-CRABP concentration versus time data using MATLAB (R2021b, MathWorks, Natick, MA, USA).

CRABP+atRA ⇌ CRABP-atRA

The differential equations for the model were solved with ode15s. Initial concentrations for CRABPs, *at*RA and holo-CRABPs were set according to experimental conditions. The association (k_on_) and dissociation (k_off_) rate constants were fitted by minimizing the sum of the difference of squares between the numerical solution of the model and the observed data with lsqcurvefit.

### 2.6. Effect of Binding Proteins on the 4-OH-atRA Formation by CYP3A4, CYP2C8, CYP26A1 and Human Liver Microsomes (HLMs)

To determine whether FABP5, CRABP1 and CRABP2 alter the metabolism of *at*RA by CYP3A4, CYP2C8 and CYP26A1, *at*RA was incubated with CYP3A4 and CYP2C8 Supersomes and with recombinant CYP26A1 insect cell microsomes reconstituted with rat P450 reductase, as previously described [17]. The formation of 4-OH-*at*RA as the metabolite of *at*RA was monitored following liquid–liquid extraction and LC-MS/MS analysis, as described previously [19,31]. In brief, at the end of the incubations, reactions were quenched with 1 mL ice-cold acetonitrile containing 10 nM of 4-oxo-*at*RA-d_3_ as an internal standard, 4 mL of ethyl acetate was added, the mixture vortexed to extract metabolites and centrifuged to separate organic and aqueous phases. The upper organic layer was transferred to a borosilicate glass tube and dried in a 25 °C water bath under gentle nitrogen flow. Samples were reconstituted in 100 µL of methanol, transferred to MS vials and analyzed by liquid chromatography-tandem mass spectrometry (LC-MS/MS) using a Sciex API5500 Q/LIT mass spectrometer (Sciex, Concord, ON, Canada) coupled to an Agilent 1290 Infinity UHPLC (Agilent Technologies, Santa Clara, CA, USA) with methods previously described [19]. Briefly, 4-OH-*at*RA was separated with a Zorbax Extend-C18 column (3.5 μm, 2.1 × 100 mm) and a mobile phase flow rate of 0.35 mL/min. Mobile phase A was water with 0.1% formic acid and mobile phase B was acetonitrile. The mobile phase gradient was as follows: 0–3 min 90% A, 10% B; then, increasing B to 50% by 3.5 min and to 85% by 7.5 min and then washing at 95% B from 7.5 to 10 min before returning to 90% A, 10% B and keeping at initial conditions until 11.5 min. The metabolites were monitored in negative ion electrospray mode and the MRM transitions used were 315 > 253 *m/z* for 4-OH-*at*RA and 316 > 272 *m/z* for 4-oxo-*at*RA-d_3_.

In initial experiments, reactions containing either 5 nM CYP3A4, 5 nM CYP2C8, 5 nM CYP26A1 reconstituted with 10 nM P450 reductase or pooled HLMs (0.2 mg/mL) were pre-incubated with 1 mM NADPH for 5 min at 37 °C in 1 mL incubation buffer (100 mM potassium phosphate, pH 7.4). The reactions were initiated by an addition of substrate, *at*RA, 1:10 *at*RA:FABP5 (CYP2C8, CYP3A4 and HLM incubations), 1:250 *at*RA:FABP5 (CYP26A1 incubations only), or 1:1 *at*RA:CRABP1, as previously described [31]. For all incubations, *at*RA was prebound with the binding proteins in the above ratios prior to the experiments. In the incubations with CYP3A4 and CYP2C8, 1 µM *at*RA was used, 20 nM *at*RA was used in experiments with CYP26A1 and 500 nM *at*RA-d_6_ was used in experiments with HLMs. CYP3A4 and CYP2C8 reactions were allowed to proceed for 10 min, HLM experiments for 30 min and CYP26A1 incubations for 2 min (to stay within linear range of product formation) before quenching the reactions with acetonitrile and extracting the metabolites for analysis, as described above.

In a separate set of experiments, CYP3A4, CYP2C8 and CYP26A1 (reconstituted with P450 reductase) were preincubated with substrate (*at*RA only, 1:1 *at*RA:CRABP1 or *at*RA:CRABP2, or 1:2 *at*RA:CRABP1 or *at*RA:CRABP2) for 5 min at 37 °C to achieve binding equilibrium prior to initiation of catalytic reactions with NADPH. In experiments with CYP3A4 and CYP2C8, 1 µM of *at*RA was used in incubations with 1:1 *at*RA:CRABP and 500 nM *at*RA was used in incubations with 1:2 *at*RA:CRABP. A total of 50 nM of *at*RA was used in incubations with CYP26A1 with 1:1 and 1:2 *at*RA:CRABP ratios. After incubation at 37 °C for 10 min for CYP3A4 and CYP2C8 and 2 min for CYP26A1, reactions were quenched with 1 mL ice-cold acetonitrile containing 10 nM 4-oxo-*at*RA-d_3_ as an internal standard and the samples were extracted and analyzed by LC-MS/MS, as described above. All incubations with CRABP1, CRABP2 and FABP5 were carried out with uncleaved, his-tagged proteins for the experiments described above.

One-way ANOVA with Dunnett’s post hoc test was used to test for differences in the 4-OH-*at*RA formation between incubations in the presence and absence of the binding proteins. A *p*-value < 0.01 was considered significant.

### 2.7. Simulations of the Impact of CRABPs on the 4-OH-atRA Formation Assuming the Free Drug Hypothesis

To test whether CRABPs function as a binding sink in the incubations, and the free drug hypothesis can explain the observed impact of CRABPs on the 4-OH-*at*RA formation by CYPs, the 4-OH-*at*RA formation as a function of free *at*RA in solution in the incubations containing CRABPs was simulated according to Equation (1):

(1) $$v=\frac{k_{cat}\times \left[at\mathrm{RA}\right]u}{K_{m}+{\left[at\mathrm{RA}\right]}_{u}}$$

In Equation (1), *v* is the velocity of the 4-OH-*at*RA formation expressed in units of pmol/min/pmol CYP, *k_cat_* is the catalytic rate constant for a given CYP of interest as measured previously and *K_m_* is the affinity of *at*RA to the CYP of interest. *K_m_* and *k_cat_* values used for CYP3A4 were 19 µM and 4 min^−1^, respectively, and the values used for CYP2C8 were 13.4 µM and 4.8 min^−1^ [19]. *K_m_* and *k_cat_* values used for CYP26A1 were determined in this study. [*at*RA]_*u*_ is the unbound concentration of *at*RA in the incubations calculated from Equation (2), as previously described [31]:

(2) $${\left[at\mathrm{RA}\right]}_{u}=\frac{\sqrt{{\left({\left[CRABP\right]}_{t}-{\left[at\mathrm{RA}\right]}_{t}+K_{d}\right)}^{2}+4K_{d}{\left[at\mathrm{RA}\right]}_{t}}-{\left[CRABP\right]}_{t}-{\left[at\mathrm{RA}\right]}_{t}+K_{d}}{2}$$

In Equation (2), [*CRABP*]*_t_* and [*at*RA]_*t*_ are the total concentrations of *CRABP* and *at*RA used in the incubations, and *K_d_* is the equilibrium dissociation constant for *at*RA with CRABP1 or CRABP2, as determined from stopped-flow experiments.

### 2.8. 4-OH-atRA Formation Kinetics with CYP26A1 in the Presence and Absence of CRABPs

To determine the effects of CRABPs on the kinetics of the 4-OH-*at*RA formation by CYP26A1, a solution of holo-CRABP1 or holo-CRABP2 was first prepared by mixing *at*RA and CRABP1 or CRABP2 in a 2:1 ratio in incubation buffer. Excess *at*RA was removed by running the solution through a spin desalting column with a 7 kDa molecular weight cutoff (Zeba, Thermo Scientific, Waltham, MA, USA). Holo-CRABP concentrations were verified with a BCA protein assay and with a fluorescence measurement against a standard curve prepared by mixing known concentrations of CRABP with 2-fold excess *at*RA. The intrinsic CRABP fluorescence for the desalted holo-CRABP and standard curve were measured as described above. In catalytic assays, 0.5 nM CYP26A1 reconstituted with 1 nM P450 reductase was preincubated with varying concentrations of *at*RA only (5–320 nM) or holo-CRABPs (5–320 nM) in 1 mL incubation buffer at 37 °C. After a 5 min preincubation, reactions were initiated with 1 mM NADPH and allowed to proceed for 2 min at 37 °C before quenching with 1 mL ice-cold acetonitrile. A total of 4 mL of ethyl acetate and 100 pmol of 4-oxo-*at*RA-d_3_ internal standard were added and the samples were extracted with ethyl acetate and 4-OH-*at*RA concentrations were analyzed by LC-MS/MS, as described above. All incubations were carried out as technical duplicates. The tight binding (Morrison) equation was fitted to the 4-OH-*at*RA formation data in Graphpad Prism 9 and the apparent unbound K_m_ (K_m,u_) and k_cat_ values were reported as the means (± S.D.) of replicate experiments conducted on three separate days.

### 2.9. Impact of Increasing CRABP to atRA Ratio on the 4-OH-atRA Formation

To determine the effect of excess apo-CRABP1 and apo-CRABP2 on the 4-OH-*at*RA formation by CYP26A1, inhibition experiments were carried out with increasing CRABP to *at*RA ratios. A total of 0.5 nM CYP26A1 reconstituted with 1 nM P450 reductase, 50 nM of *at*RA and 0–400 nM CRABP1 or CRABP2 were preincubated for 5 min in 1 mL incubation buffer at 37 °C before initiation of catalysis with 1 mM NADPH (final concentration). Reactions were quenched with 1 mL ice-cold acetonitrile after 2 min. A total of 4 mL of ethyl acetate and 100 pmol of 4oxo-*at*RA-d_3_ were added and 4-OH-*at*RA was extracted and analyzed via LC-MS/MS, as described in Section 2.6. To ensure the correct ratios of *at*RA:CRABP were used, holo-CRABPs were prepared with spin desalting columns, as described in Section 2.8. For incubations with excess *at*RA, holo-CRABPs were diluted to 0–40 nM and *at*RA was added to 50 nM total *at*RA. For incubations with excess CRABP, holo-CRABPs were diluted to 50 nM and apo-CRABP was added for final concentrations of 50–400 nM total CRABP. Experiments were carried out in technical duplicates on three separate days for CRABP1 and two separate days for CRABP2.

### 2.10. Analysis of the Kinetics of Protein–Protein Interactions between CYP26A1, CYP26B1 and CRABPs

It has been previously suggested that CRABP1 and CRABP2 interact directly with CYP26B1 both via apo-CRABPs inhibiting CYP26B1 activity and via holo-CRABPs directly channeling *at*RA for metabolism with CYP26B1 [31]. To test whether similar protein–protein interactions can explain the kinetics of the 4-OH-*at*RA formation by CYP26A1 in the presence of CRABP1 and CRABP2, the previously described Equation (3) [31] was fitted to the combined 4-OH-*at*RA formation data from the experiments described in Section 2.8:

(3) $$v=\frac{k_{cat}{\left[at\mathrm{RA}\right]}_{u}\left(1+\frac{\beta {\left[CRABP\right]}_{u}}{\alpha K_{d}}\right)}{K_{m}\left(1+\frac{{\left[CRABP\right]}_{u}}{K_{i}}\right)+{\left[at\mathrm{RA}\right]}_{u}\left(1+\frac{{\left[CRABP\right]}_{u}}{\alpha K_{d}}\right)}\;\;$$

In Equation (3), *v* is the velocity of the 4-OH-*at*RA formation in the presence of CRABP expressed in units of pmol/min/pmol CYP; *k_cat_* is the rate of catalysis for the 4-OH-*at*RA formation by the CYP26 in the absence of CRABP; [*at*RA]_*u*_ and [*CRABP*]_*u*_ are the concentrations for unbound *at*RA and unbound CRABP, respectively; *K_m_* is the Michaelis–Menten constant for the 4-OH-*at*RA formation in the absence of CRABP; *α* and *β* are model parameters to describe the effect of CRABP on *K_m_* and *k_cat_*, respectively; *K_i_* is the affinity of apo-CRABP to CYP; and *K_d_* is the binding affinity of *at*RA to the specific CRABP. The derivation for Equation (3) is provided in Supplementary Materials. The unbound *at*RA and CRABP concentrations were calculated according to Equation (2) under the assumption that concentrations of the CYP-RA, CRABP-CYP and CRABP-CYP-RA complexes were negligible in comparison to *at*RA and CRABP concentrations in the experiments. Observed 4-OH-*at*RA formation velocities were fit globally with the fixed *K_m_* and *k_cat_* values determined in this study in incubations without CRABPs, and *K_d_* values were fixed to values determined from stopped-flow experiments confirmed with fluorescence titrations.

## 3. Results

### 3.1. FABP5 Does Not Affect atRA Hydroxylation by CYPs

**Figure 1:**
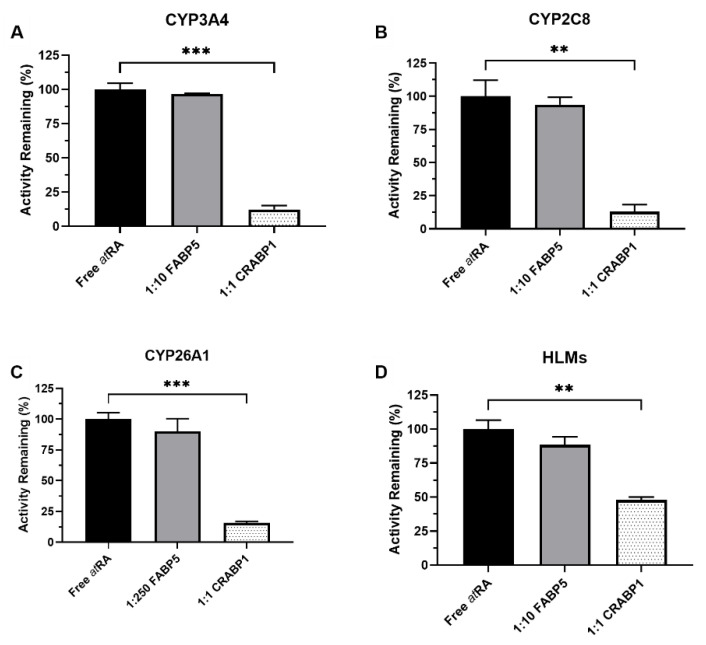
Effect of FABP5 and CRABP1 on the metabolism of *at*RA. The 4-OH-*at*RA formation was measured in incubations with recombinant CYPs and HLMs in the presence and absence of binding proteins. The incubations were conducted, as described in Materials and Methods, with all reactions initiated by adding the substrate pre-complexed with the binding protein. The impact of FABP5 (10 or 250-fold excess to *at*RA) and CRABP1 (equal concentrations as *at*RA) on 4-OH-*at*RA formation by (**A**) CYP3A4, (**B**) CYP2C8, (**C**) CYP26A1 and (**D**) human liver microsomes (HLMs) is shown. *at*RA concentration was 1 µM in (**A**,**B**), 20 nM in (**C**) and 500 nM in (**D**). ** *p*-value < 0.01, *** *p*-value < 0.001, one-way ANOVA with Dunnett’s post hoc test.

FABP5 has been previously shown to facilitate *at*RA-induced PPARβ/δ signaling [29]. However, it is not known whether FABP5 affects *at*RA metabolism by CYPs. The impact of FABP5 on the 4-OH-*at*RA formation by CYPs previously shown to metabolize *at*RA [20] was evaluated in comparison to the effect by CRABP1. FABP5 in 10-fold excess to *at*RA had no effect on the 4-OH-*at*RA formation by CYP3A4 (97 ± 0.5% activity remaining) or CYP2C8 (94 ± 6% activity remaining) (Figure 1A,B). In contrast, CRABP1 (in 1:1 ratio to *at*RA) decreased 4-OH-*at*RA formation by CYP3A4 and CYP2C8 by 88 ± 3% and 87± 5% (*p*-value < 0.01), respectively, in comparison to free *at*RA as a substrate (Figure 1). FABP5 did not alter the 4-OH-*at*RA formation by CYP26A1 either when *at*RA was incubated in the presence of 250-fold excess of FABP5. The 4-OH-*at*RA formation by CYP26A1 was 90 ± 10% of control in the presence of FABP5 and not significantly (*p-*value > 0.01) different from the 4-OH-*at*RA formation from free *at*RA. In comparison, the 4-OH-*at*RA formation was significantly (*p*-value < 0.001) reduced to 16 ± 1% of the free *at*RA control in the presence of CRABP1 (Figure 1C). Furthermore, a 10-fold excess of FABP5 had little effect on the 4-OH-*at*RA formation in pooled HLMs compared to CRABP1 (1:1) (88 ± 6% vs. 48 ± 2% activity remaining) (Figure 1D). Taken together, these results suggest that FABP5 does not sequester *at*RA to prevent metabolism by CYPs while CRABP1 has a significant effect on *at*RA metabolism by CYPs including CYP26A1.

### 3.2. atRA Binds CRABPs with Nanomolar Affinity

**Figure 2:**
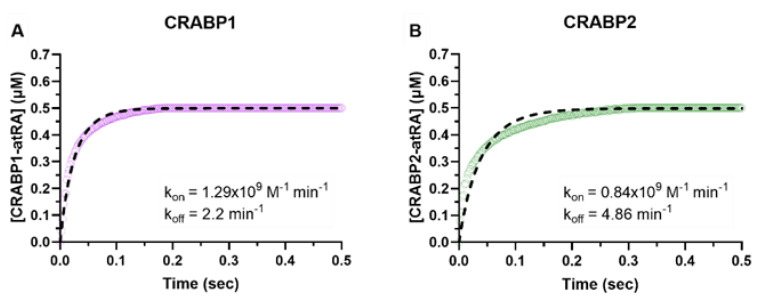
CRABP–*at*RA-binding kinetics determined with stopped-flow. Association of *at*RA (2 μM) with CRABP1 (**A**) and CRABP2 (**B**) (0.5 μM) was monitored from the increase in *at*RA fluorescence due to CRABP binding, as described in Materials and Methods. The concentration of holo-CRABP was calculated based on the fluorescence yield of holo-CRABP. The shaded line in panels (**A**,**B**) show the observed time course of change in holo-CRABP1 (**A**) and holo-CRABP2 (**B**) concentrations following mixing of CRABPs with *at*RA. The dashed line shows the model fits to the data. The fitted association (k_on_) and dissociation (k_off_) constants for the specific experiment are shown in insets.

The binding kinetics for *at*RA with CRABP1 and CRABP2 were determined with stopped-flow (Figure 2) and the k_on_ and k_off_ were estimated from a 2-state binding model (Materials and Methods*)*. The k_on_ and k_off_ values and the calculated equilibrium dissociation constants (K_d,_ 4.7 ± 3.8 nM and 7.6 ± 4.0 nM for CRABP1 and CRABP2, respectively) from three replicate experiments were averaged and the mean values are shown in Table 1.

### 3.3. CRABPs Sequester atRA from CYP3A4 and CYP2C8 as Predicted by the Free Drug Hypothesis

**Figure 3:**
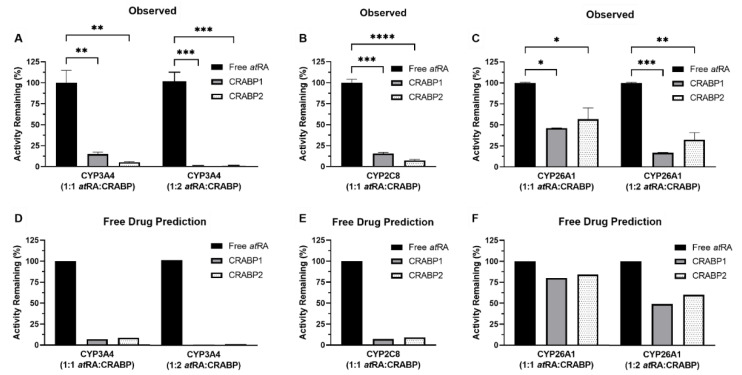
The 4-OH-*at*RA formation by CYP3A4, CYP2C8 and CYP26A1 in the presence of CRABPs. The impact of CRABPs on the 4-OH-*at*RA formation by CYP3A4, CYP2C8 and CYP26A1 was assessed in incubations with recombinant enzymes. At a 1:1 ratio of CRABP to *at*RA, both CRABP1 and CRABP2 decreased the 4-OH-*at*RA formation by CYP3A4 (**A**) (to 15 ± 2% and 5 ± 0.7% of control, respectively) and CYP2C8 (**B**) (to 16 ± 2% and 7 ± 1% of control, respectively), and completely abolished CYP3A4 activity at a 1:2 *at*RA to CRABP ratio (**A**) (>99% decrease). The observed activity of CYP3A4 and CYP2C8 in the presence of CRABPs is consistent with predictions made via calculating the unbound concentrations of *at*RA (**D**,**E**) present in the incubations based on the experimental K_d_ values from stopped-flow experiments. (**C**) The 4-OH-*at*RA formation by CYP26A1 was decreased by about 50% at a 1:1 ratio of CRABP to *at*RA and > 70% in the presence of 2-fold excess CRABPs. Calculated unbound concentrations of *at*RA predicted higher 4-OH-*at*RA formation by CYP26A1 (**F**) than observed, suggesting that the free drug hypothesis cannot explain CYP26A1 activity in the presence of CRABPs. (* *p*-value < 0.05, ** *p*-value < 0.01, *** *p*-value < 0.001, **** *p*-value < 0.0001, one-way ANOVA with Dunnett’s post hoc test).

The binding of *at*RA with CRABP1 or CRABP2 has been shown to decrease the 4-OH-*at*RA formation by CYP3A4 and CYP2C8 [31], likely due to sequestration of *at*RA from metabolic enzymes according to the free drug hypothesis. To test this hypothesis, *at*RA was incubated with CYP3A4, CYP2C8 and CYP26A1 in the presence and absence of equimolar concentrations of CRABP1 and CRABP2 or a 2-fold excess of CRABPs, and the 4-OH-*at*RA formation velocities were measured following the initiation of the catalytic reactions with the addition of NADPH. In the presence of equimolar concentrations of CRABP1 or CRABP2 with *at*RA, CRABP1 decreased the 4-OH-*at*RA formation by CYP3A4 and CYP2C8 by about 85% and CRABP2 decreased the 4-OH-*at*RA formation by about 95% when compared to no CRABP present (Figure 3A,B, *p*-value < 0.01). The 4-OH-*at*RA formation by CYP3A4 was completely abolished in the presence of 2-fold excess CRABP (>99%, *p*-value < 0.001, Figure 3A). CRABP1 and CRABP2 decreased the 4-OH-*at*RA formation in incubations with CYP26A1 by about 50% with equimolar concentrations of CRABP and *at*RA compared to incubations with no CRABP (*p*-value < 0.05, Figure 3C). The 4-OH-*at*RA formation was further decreased to about 20% and 30% of no CRABP controls with 2-fold excess CRABP1 and CRABP2, respectively (*p*-value < 0.01, Figure 3C).

To test whether these effects could be explained by the free drug hypothesis (i.e., *at*RA being sequestered to CRABPs and only free *at*RA being available for metabolism by CYPs), the 4-OH-*at*RA formation by the CYPs in the presence of CRABPs was simulated with unbound concentrations of *at*RA calculated with the binding kinetics of *at*RA to CRABPs determined using stopped-flow, and assuming that CRABPs would not interact with the CYPs. The free drug hypothesis predicted a 93% and 91% decrease in the 4-OH-*at*RA formation by both CYP3A4 and CYP2C8 in the presence of equimolar concentrations of CRABP1 and CRABP2, respectively, when compared to free *at*RA, while a 2-fold excess of CRABPs was predicted to eliminate nearly all 4-OH-*at*RA formation by CYP3A4 and CYP2C8 (>99%, Figure 3D) due to binding of free *at*RA to CRABPs. These predictions were in good agreement with observed values and are consistent with the notion that CRABPs bind *at*RA tightly to sequester *at*RA from metabolism. In contrast, the free drug hypothesis over-predicted the 4-OH-*at*RA formation by CYP26A1 compared to the observed values. The 4-OH-*at*RA formation was predicted to decrease by about 20% in the presence of equimolar concentrations of CRABP1 and CRABP2, and by 50% and 40% with 2-fold excess CRABP1 and CRABP2, respectively. These predictions suggest that, unlike CYP3A4 and CYP2C8 activity, the binding proteins have an additional inhibitory effect on CYP26A1 activity in addition to binding *at*RA.

### 3.4. Determination of the Kinetics of the 4-OH-atRA Formation by CYP26A1 in the Presence of CRABPs

**Figure 4:**
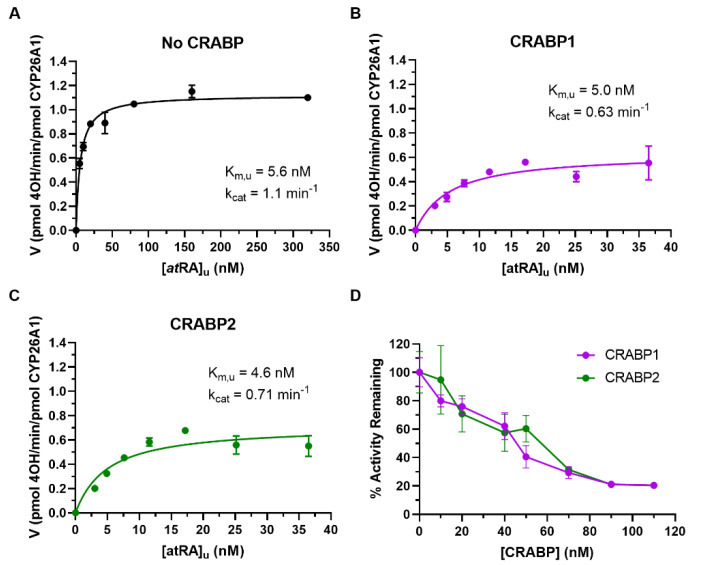
The 4-OH-*at*RA formation kinetics by CYP26A1 in the presence of CRABPs. Varying concentrations of holo-CRABP1 and holo-CRABP2 (5–320 nM) were incubated with recombinant CYP26A1, as described in *Material and Methods*. (**A**–**C**) Representative experiments of the 4-OH-*at*RA formation by CYP26A1 in the presence and absence of CRABPs. The Morrison tight binding equation was fitted to the data and the unbound K_m_ and k_cat_ values are shown in the inset for the representative experiment shown. (**D**) Inhibition of theCYP26A1 mediated 4-OH-*at*RA formation by CRABPs is shown with increasing CRABP1 and CRABP2 concentrations relative to *at*RA (50 nM).

To define the kinetics of the 4-OH-*at*RA formation by CYP26A1 in the presence of CRABP1 and CRABP2, varying concentrations of holo-CRABPs or free *at*RA were incubated with recombinant CYP26A1 and the 4-OH-*at*RA formation measured (Figure 4A–C). The apparent K_m_ values of the 4-OH-*at*RA formation increased in the presence of CRABP1 (11.7 ± 3.44 nM) and CRABP2 (9.7 ± 3.2 nM) when compared to *at*RA alone (4.7 ± 0.81 nM) but the unbound K_m_ values were unchanged in the presence of CRABPs (Figure 4). The apparent k_cat_ values decreased in the presence of CRABP1 (0.71 ± 0.07 min^−1^) and CRABP2 (0.75 ± 0.09 min^−1^) when compared to the k_cat_ with *at*RA alone (1.07 ± 0.08 min^−1^). While the finding of the unchanged unbound K_m_ is consistent with the free drug hypothesis, the decrease in k_cat_ cannot be explained via simple Michaelis–Menten kinetics.

Previous studies with CYP26B1 have suggested that CRABP1 and CRABP2 interact directly with CYP26B1, and apo-CRABPs were shown to inhibit the 4-OH-*at*RA formation by CYP26B1. To test whether apo-CRABPs inhibit *at*RA metabolism by CYP26A1 as well, CYP26A1 was incubated with *at*RA in the presence of increasing concentrations of CRABP. Increasing concentrations of apo-CRABPs decreased the 4-OH-*at*RA formation by CYP26A1 (Figure 4D), suggesting that CRABPs may directly interact with CYP26A1.

**Figure 5:**
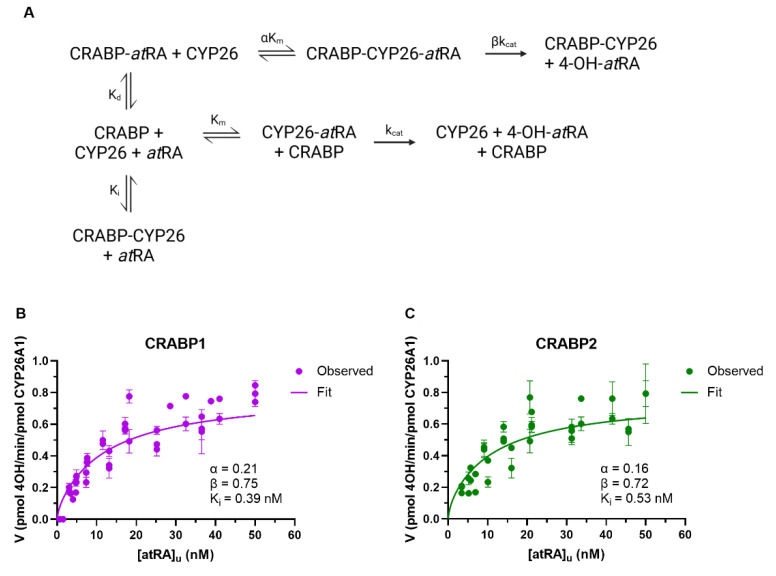
The CRABP-CYP26 interaction model and fit of the interaction model to the 4-OH-*at*RA formation data. The kinetic scheme for the model of the 4-OH-*at*RA formation by CYP26s in the presence of CRABPs that incorporates holo-CRABP and apo-CRABP-CYP26 interactions is shown in (**A**) (created with BioRender.com accessed on 8 April 2022). αK_m_ is the affinity of holo-CRABP for CYP26, K_i_ is the affinity of apo-CRABP for CYP26 and βk_cat_ is the rate of catalysis for an *at*RA-CRABP-CYP26 ternary complex. The model was globally fit to the data with CYP26A1 as described in Materials and Methods, and plots with observed 4-OH-*at*RA formation data (circles) and model fits (solid lines) for CYP26A1 in the presence of CRABP1 (**A**) and CRABP2 (**B**) are shown.

Increasing concentrations of CRABPs beyond *at*RA concentrations result in a decrease in free concentration of *at*RA in the incubations in addition to potentially inhibiting the activity of CYP26A1 via direct protein–protein interactions. To define the kinetic constants of CRABP–CYP26A1 interactions, a previously described model incorporating protein–protein interactions between CRABPs and CYP26A1 (Figure 5A) was fitted to the data of the 4-OH-*at*RA formation by CYP26A1 in the presence of increasing concentrations of CRABPs. Based on model parameter fits (Figure 5B,C), apo-CRABP1 and apo-CRABP2 inhibit CYP26A1 (K_i_ = 0.39 nM and 0.53 nM, for CRABP1 and CRABP2, respectively). In addition, holo-CRABP1 and holo-CRABP2 had higher affinities to CYP26A1 than free *at*RA (αK_m_ = 0.99 nM and 0.75 nM for CRABP1 and CRABP2, respectively), while the catalytic rate was lower from the ternary complex than from the *at*RA–CYP26A1 complex (βk_cat_ = 0.80 min^−1^ and 0.77 min^−1^, respectively).

## 4. Discussion

Cellular *at*RA concentrations are regulated by a network of enzymes that catalyze the oxidation of retinol to *at*RA and the clearance of *at*RA. Tight regulation of *at*RA concentrations is necessary for cellular health and for biological processes, as even small, 2- to 5-fold changes in retinoid concentrations alter *at*RA signaling [5,6,36]. Notably, it has been shown that short duration pulses of altered *at*RA concentrations likely trigger cyclic spermatogenesis, and gradients of *at*RA concentrations are established across the seminiferous epithelium to control cell differentiation [36]. Despite the critical function of such *at*RA gradients in regulating cellular processes, the mechanisms that regulate the establishment of these critical gradients and pulses of increased *at*RA concentrations have not been established. Although the CYP26 enzymes CYP26A1 and CYP26B1 and the ALDH1A enzymes are the main enzymes responsible for *at*RA clearance and synthesis during embryonic development and adult life [14,37], neither CYP26 nor ALDH1A enzyme expression changed across the spermatogenic cycle in the mouse testis [36]. This suggests that other mechanisms are present to regulate rapid changes and pulses of *at*RA concentrations, essential for retinoid signaling. One such potential signal is the change in CRABP1 expression across the spermatogenic cycle [36] that may regulate *at*RA clearance in a stage-specific manner via functioning as a regulator of *at*RA clearance through protein–protein interactions with the CYP26 enzymes. The data shown here are consistent with a mechanism in which the changes in CRABP1 and CRABP2 expression regulate *at*RA clearance and 4-OH-*at*RA formation via direct interactions with CYP26 enzymes to allow rapid changes in cellular *at*RA concentrations without altering CYP26 and ALDH1A expressions.

The CRABPs have been previously shown to have distinct effects on *at*RA 4-hydroxylation by CYP26B1 and CYP26C1 [19,31] and *at*RA desaturation by CYP27C1 [32]. In addition, *at*RA oxidation by CYP3A4 and CYP2C8 is completely abolished in the presence of CRABPs, a finding consistent with the free drug hypothesis and tight binding of *at*RA with CRABPs. CRABP1 and CRABP2 binding decreased the apparent K_m_ values (determined from fitting the Michaelis–Menten model to total *at*RA concentration versus product formation) of *at*RA with CYP26B1 [31], while the apparent K_m_ values of *at*RA hydroxylation were similar for CYP26C1 in the presence and absence of CRABP1 and CRABP2 [19]. For *at*RA desaturation by CYP27C1, both CRABP1 and CRABP2 increased the apparent K_m_ values [32]. In this study, the apparent K_m_ value of *at*RA with CYP26A1 increased in the presence of CRABPs, an effect distinctly different from previous observations with CYP26B1. However, the unbound K_m_ values for *at*RA with CYP26A1 were not different in the presence of CRABPs, a finding supporting an interpretation that only free *at*RA interacts with CYP26A1 to undergo oxidation. Yet, CRABP1 and CRABP2 decreased the apparent k_cat_ value of *at*RA hydroxylation by CYP26A1, similar to prior findings with CYP26B1, CYP26C1 and rat testis microsomes [15,16,19,31] and of *at*RA desaturation by CYP27C1 [32]. With CYP26B1 and CYP27C1, CRABP1 had a greater effect on k_cat_ values than CRABP2 while, with CYP26C1 and CYP26A1, the decrease in k_cat_ values for *at*RA 4-hydroxylation were similar with both CRABPs. The decrease in the apparent k_cat_ value has previously been explained via a protein–protein interaction between CRABPs and CYP26 enzymes, with apo-CRABPs inhibiting the CYP26B1 mediated hydroxylation of *at*RA. Similarly, apo-CRABPs were found to inhibit the desaturation of *at*RA by CYP27C1 [32]. The differences in the effects on apparent k_cat_ values with the different CYP enzymes are likely due to specific and unique allosteric interactions between the CRABP1 and CRABP2 and the specific CYP enzymes. Further studies to identify the interacting residues between CRABPs and CYPs are needed to define the mechanisms of the protein–protein interactions.

Analyses of the kinetics of the protein–protein interactions between CRABPs and CYPs hinge on the knowledge of the binding kinetics of *at*RA with CRABP1 and CRABP2. The K_d_ values of *at*RA with CRABPs are typically measured with fluorescence titrations under equilibrium assumptions yielding K_d_ values ranging from 0.4–16 nM for CRABP1 and 2–39 nM for CRABP2 [23,25,26,27]. Stopped-flow was previously used to measure the k_on_ and k_off_ for *at*RA binding with CRABPs [24]. These studies used a large lipid sink to trap dissociated *at*RA and a simple monoexponential was fit to the dissociation data to estimate k_off_. The resulting K_d_ estimates (0.06 nM and 0.13 nM for CRABP1 and CRABP2, respectively) were considerably lower than those measured by fluorescence titrations or by stopped-flow here, likely due to the differences in the model fit to the data. When the previously published data was analyzed fitting the same model to the data as shown here, the K_d_ value for CRABP1 was 2.2–5.5 nM and for CRABP2 2.6–15 nM. The k_on_ ranged from 2.7 × 10^6^ (dissociation experiment) to 1.9 × 10^9^ (association experiment) M^−1^ min^−1^ for CRABP1 and from 9.4 × 10^6^ (dissociation experiment) to 1.8 × 10^9^ (association experiment) M^−1^ min^−1^ for CRABP2. For k_off_ the values ranged from 0.015 (dissociation experiment) to 4.7 (association experiment) min^−1^ for CRABP1 and from 0.025 (dissociation experiment) to 28 (association experiment) min^−1^ for CRABP2, demonstrating the low confidence in the individual kinetic constants. A simple monoexponential fit as used previously fails to account for the simultaneous binding and dissociation processes that occur during the stopped-flow experiments resulting in inaccurate estimates for the kinetic parameters. In addition, the lipid sink likely effects some of the fluorescence data and kinetics of binding in the dissociation experiment. To explore whether the kinetics of *at*RA binding with CRABPs reported previously could be replicated, single concentration stopped-flow experiments were undertaken in this study. The k_on_ values for CRABP1 and CRABP2 were found to be similar to what was previously reported, but the k_off_ was found to be substantially faster (4.4 min^−1^ and 7.9 min^−1^ for CRABP1 and CRABP2, respectively) than previously reported but consistent with the previous data when the same model was used to analyze both datasets. The resulting K_d_ values (4.7 nM and 7.60 nM for CRABP1 and CRABP2, respectively) are similar to those measured via fluorescence titrations (0.4–16 nM and 2–39 nM for CRABP1 and CRABP2, respectively) [23,25,26,27] and those calculated here from the previously published stopped-flow data [24]. A limitation of these experiments is that we and the previous publication only tested the binding kinetics at a single concentration of *at*RA and CRABP and further experiments are needed to fully characterize the binding kinetics (individual k_on_ and k_off_ values) and the impact of conformational changes on the binding kinetics including the most appropriate kinetic model to fit to the data.

**Figure 6:**
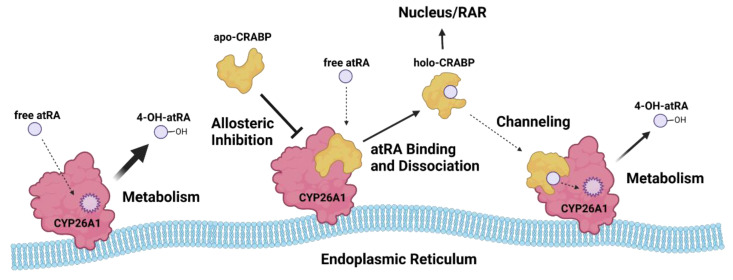
Kinetics informed model of CRABP cellular functions in regulating *at*RA metabolism and homeostasis. CYP26A1 metabolizes *at*RA to 4-OH-*at*RA to facilitate *at*RA clearance from the cell. Apo-CRABP is bound to CYP26A1 in the ER to prevent *at*RA metabolism when *at*RA levels are scarce and an excess of apo-CRABP is present. When *at*RA is abundant, more CRABP is bound to *at*RA, releasing holo-CRABP from CYP26A1 and increasing holo-CRABP levels in the cell. Holo-CRABP may facilitate the delivery of *at*RA to nuclear receptors or directly channel *at*RA to CYP26A1 for metabolism (figure created with BioRender.com accessed on 8 April 2022).

The determination of the K_d_ for *at*RA with CRABPs allowed fitting of the complete model of CRABP–CYP26A1 protein–protein interactions and the 4-OH-*at*RA formation by CYP26A1 (Figure 5A) [31] globally to the 4-OH-*at*RA formation data in the presence of different concentrations of CRABP1 and CRABP2. Fitting the model to the data established that apo-CRABP1 and apo-CRABP2 bind to CYP26A1 with high affinity. As suggested previously for both CYP26B1 and CYP27C1, CRABP1 and CRABP2 appear to also channel *at*RA for metabolism by CYP26A1 with the observed catalytic rate (βk_cat_) somewhat slower (0.8 min^−1^) in the presence of CRABPs than in their absence (1.1 min^−1^) either due to slow off-rate of *at*RA from CRABP to CYP26A1 via the ternary CRABP–*at*RA–CYP26A1 complex or due to a lower catalytic rate of the 4-OH-*at*RA formation by the ternary complex. The current kinetic analyses do not allow the differentiation of these mechanistic possibilities. The parameter estimates suggest that apo-CRABPs have a slightly higher affinity to CYP26A1 than holo-CRABPs. The αK_m_ of holo-CRABP1 was 0.99 nM in comparison to the K_i_ of apo-CRABP1 of 0.39 nM with CYP26A1. Similarly, the αK_m_ of holo-CRABP2 with CYP26A1 was 0.75 nM while the K_i_ of apo-CRABP2 was 0.53 nM with CYP26A1. Taken together, this suggests that CRABP-CYP interactions alter the rate of *at*RA metabolism via a network of protein–protein interactions that involve both the inhibition of metabolism by apo-CRABP and channeling of *at*RA for metabolism via protein–protein interactions of holo-CRABP (Figure 6).

The high affinity of CRABPs with CYP26s is consistent with the observations by fluorescence microscopy, showing that CRABPs localize to the ER membrane [30]. The data shown here suggest that this localization is driven by the affinity of apo-CRABPs to the CYP26 enzymes expressed in the ER and allow the development of a model (Figure 6) of the overall regulation of *at*RA metabolism and CYP26A1 activity by CRABPs. This is consistent with a previous suggestion that CRABPs may play a role in modulating *at*RA concentrations in the cells by fine-tuning *at*RA clearance at different *at*RA concentrations via interactions with CYP enzymes in the ER [15,16,22,31,37]. It is notable that the affinity of *at*RA (K_d_) to CRABPs is similar to the cellular concentration of *at*RA (5–10 nM) in mammals [21]. For example, if CRABP concentrations in the cells are around 5 nM, at low *at*RA concentrations (1–5 nM), the majority of CRABP would be in the apo-CRABP form and inhibit CYP26A1 activity to preserve free *at*RA, while at high *at*RA concentrations (10–20 nM), nearly all CRABP would be bound with *at*RA and the holo-CRABP would act predominantly via channeling *at*RA for metabolism, bringing *at*RA concentrations back to the desired concentrations rapidly. Similarly, under the circumstances that CRABP expression levels change, a lower CRABP expression would ensure higher free *at*RA concentrations in the cell, potentially allowing for pulses of *at*RA concentrations to be established prior to *at*RA getting cleared effectively by CYP26s. Similarly, high CRABP expression levels would sequester free *at*RA and inhibit *at*RA clearance by CYP26s, preventing further metabolism of *at*RA when *at*RA is scarce. It is noteworthy that the interactions of CRABP1 and CRABP2 with CYP26A1 appear kinetically very similar. However, only CRABP2 translocates to the nucleus upon *at*RA-binding and channels *at*RA to RAR while CRABP1 is restricted to cytosolic and ER localization. As such, in the context of CRABP1, low CRABP1 expression would liberate free *at*RA for signaling while low CRABP2 may facilitate *at*RA metabolism. Further studies of the cellular network of CRABP interactions and localization are needed to fully elucidate the mechanisms on how the CRABP expression affects retinoid signaling.

In addition to CRABPs, FABP5 has been shown to bind *at*RA with a K_d_ of 57 nM [29] and FABP5 has been suggested to modulate *at*RA signaling in cells. Hence, we also assessed whether FABP5 binding would impact *at*RA metabolism similar to CRABPs. No effect of FABP5 on *at*RA metabolism was observed in incubations with CYP3A4, CYP2C8, CYP26A1 or HLMs, suggesting that the impact of FABP5 on *at*RA homeostasis is limited to effects on nuclear receptor signaling in select cell types. It is also possible that the binding affinity of *at*RA to human FABP5 is lower than that measured previously [29], resulting in insignificant binding of *at*RA to FABP5 in the incubations.

In conclusion, apo- and holo-CRABP1 and CRABP2 interact with CYP26A1 with high affinity and alter the metabolism of *at*RA by CYP26A1. The data collected support a model where CRABPs serve as a switch to regulate *at*RA concentrations and signaling in cells dictated by the ratio of apo- to holo-CRABP. The apo-CRABPs appear to inhibit the clearance of *at*RA, while holo-CRABPs likely play a dual role of channeling *at*RA to nuclear receptors and to CYP26 enzymes. Taken together, these results suggest that cellular *at*RA signaling and *at*RA gradients and pulses may be modulated by temporary changes in the CRABP expression.

## Figures and Tables

**Table 1:** CRABP–*at*RA Binding Kinetics as Measured by Stopped-Flow.

	k_on_ (M^−1^ min^−1^)	k_off_ (min^−1^)	K_d_ (nM)
CRABP1	1.07 × 10^9^ ± 2.7 × 10^8^	4.40 ± 2.4	4.7 ± 3.8
CRABP2	0.96 × 10^9^ ± 2.2 × 10^8^	7.89 ± 6.0	7.6 ± 4.0

Data reported as means ± S.D. from three separate experiments. Due to limited stopped-flow data the K_d_ value cannot be solely determined from these experiments with confidence and should be considered together with published [22] K_d_ values.

## Data Availability

All raw data, model fits and MATLAB code are available upon request from the corresponding author.

## Supplementary Materials

The following supporting information can be downloaded at: https://www.mdpi.com/article/10.3390/nu14091784/s1, Figure S1: Plasmid maps and coding sequences for CRABP1, CRABP2 and FABP5 clones in pET28a+ expression vectors.
