# Supplementary material for: CRABPs Alter all-trans-Retinoic Acid Metabolism by CYP26A1 via Protein-Protein Interactions
Source: Nutrients. 2022 Apr 24;14(9):1784. doi: 10.3390/nu14091784 (PMC9105409; doi:10.3390/nu14091784)
Supplement: Supplementary file 1 [file nutrients-14-01784-s001.zip › nutrients-1666415-supplementary.pdf]

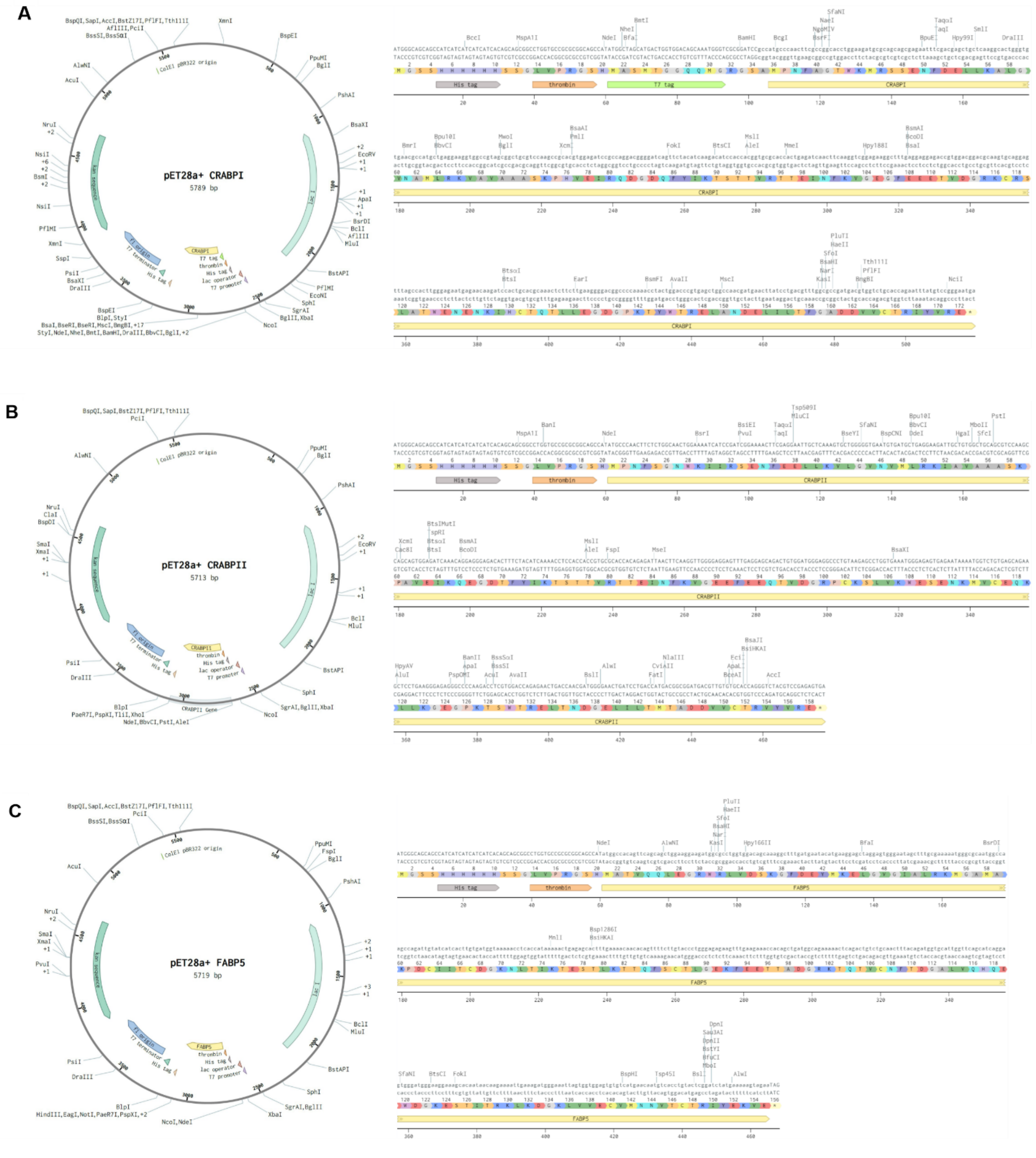

Figure S1: Plasmid maps and coding sequences for CRABP1, CRABP2, and FABP5 clones in pET28a+ ex-pression vectors. Benchling [Biology Software]. (2022). Retrieved from https://benchling.com.

Derivation of the steady-state velocity equation for 4OH-*at*RA formation in the presence of CRABP-CYP interactions (equation 3).

The steady-state velocity equation was derived according to the standard procedures for deriving velocity equations for rapid equilibrium systems including Michaelis-Menten described in *Segel. 1975. Enzyme Kinetics: Behavior and Analysis of Rapid Equilibrium and Steady-State Enzyme Systems, p. 18-24*. Based on the kinetic scheme in Figure 5A, the velocity dependence equation for 4-OH-*at*RA formation can be described as:

$$v = k_{cat}[atRA - CYP] + \beta k_{cat}[CRABP - atRA - CYP]$$

Both sides of the velocity dependence equation are divided by $[CYP]_t$ where on the right hand side $[CYP]_t$ is written as the sum of all CYP species:

$$\frac{v}{[CYP]_t} = \frac{k_{cat}[atRA - CYP] + \beta k_{cat}[CRABP - atRA - CYP]}{[CYP]_u + [atRA - CYP] + [CRABP - CYP] + [CRABP - atRA - CYP]}$$

Given the definitions of the equilibrium binding constants:

$$K_m = \frac{[atRA]_u[CYP]_u}{[atRA - CYP]}$$

$$K_d = \frac{[atRA]_u[CRABP]_u}{[atRA - CRABP]}$$

$$K_i = \frac{[CRABP]_u[CYP]_u}{[CRABP - CYP]}$$

$$\alpha K_m = \frac{[CRABP - atRA][CYP]_u}{[CRABP - atRA - CYP]}$$

each term in the velocity dependence equation can be expressed in terms of $[CYP]_u$:

$$\frac{v}{[CYP]_t} = \frac{k_{cat}\frac{[atRA]_u[CYP]_u}{K_m} + \beta k_{cat}\frac{[CRABP]_u[atRA]_u[CYP]_u}{\alpha K_m K_d}}{[CYP]_u + \frac{[atRA]_u[CYP]_u}{K_m} + \frac{[CRABP]_u[CYP]_u}{K_i} + \frac{[CRABP]_u[atRA]_u[CYP]_u}{\alpha K_m K_d}}$$

and rearranged to:

$$\frac{v}{[CYP]_t} = \frac{[CYP]_u(k_{cat}\frac{[atRA]_u}{K_m} + \beta k_{cat}\frac{[CRABP]_u[atRA]_u}{\alpha K_m K_d})}{[CYP]_u(1 + \frac{[atRA]_u}{K_m} + \frac{[CRABP]_u}{K_i} + \frac{[CRABP]_u[atRA]_u}{\alpha K_m K_d})}$$

Cancelling $[CYP]_u$:

$$\frac{v}{[CYP]_t} = \frac{k_{cat}(\frac{[atRA]_u}{K_m} + \frac{\beta[CRABP]_u[atRA]_u}{\alpha K_m K_d})}{1 + \frac{[atRA]_u}{K_m} + \frac{[CRABP]_u}{K_i} + \frac{[CRABP]_u[atRA]_u}{\alpha K_m K_d}}$$

Since $V_{max} = k_{cat} * [CYP]_t$, the velocity dependence equation can be expressed as:

$$v = \frac{V_{max}\,(\frac{[atRA]_u}{K_m} + \frac{\beta[CRABP]_u[atRA]_u}{\alpha K_m K_d})}{1 + \frac{[atRA]_u}{K_m} + \frac{[CRABP]_u}{K_i} + \frac{[CRABP]_u[atRA]_u}{\alpha K_m K_d}}$$

Simplifying top and bottom terms:

$$v = \frac{V_{max}\left([atRA]_u + \frac{\beta[CRABP]_u[atRA]_u}{\alpha K_d}\right)}{K_m + [atRA]_u + \frac{K_m[CRABP]_u}{K_i} + \frac{[CRABP]_u[atRA]_u}{\alpha K_d}}$$

$$v = \frac{V_{max}[atRA]_u\left(1 + \frac{\beta[CRABP]_u}{\alpha K_d}\right)}{K_m\left(1 + \frac{[CRABP]_u}{K_i}\right) + [atRA]_u\left(1 + \frac{[CRABP]_u}{\alpha K_d}\right)}$$

Since v is normalized to experimental $[CYP]_t$ used in incubations (i.e. units of v = pmol/min/pmol CYP), then the velocity equation to describe the data becomes equation 3:

$$v = \frac{k_{cat}[atRA]_u\left(1 + \frac{\beta[CRABP]_u}{\alpha K_d}\right)}{K_m\left(1 + \frac{[CRABP]_u}{K_i}\right) + [atRA]_u\left(1 + \frac{[CRABP]_u}{\alpha K_d}\right)}$$
